# Questions as data: illuminating the potential of learning analytics through questioning an emergent field

**DOI:** 10.1186/s41039-016-0037-1

**Published:** 2016-05-21

**Authors:** Jon Mason, Weiqin Chen, Tore Hoel

**Affiliations:** 1 School of Education, Charles Darwin University, Darwin, 0909 NT Australia; 2 Oslo and Akershus University College of Applied Sciences, Oslo, Norway

**Keywords:** Learning analytics, Question formulation, Interoperability, Research agenda

## Abstract

In providing a meta-analysis of a series of workshop papers and questions arising on the emergent field of learning analytics, this paper contributes to the ongoing formation of a shared research agenda. The first ICCE Learning Analytics workshop in 2014 demonstrated the effectiveness of a focused questioning session for collecting relevant data beyond the content of the papers themselves. In December 2014, approximately 40 participants attended the workshop held in Nara, Japan, and contributed to the collection of open research questions. Six papers were presented covering topics including scope; interoperability standards; privacy and control of individual data, extracting data from learning content and processes; and the development of conceptual frameworks. These papers established a base from which the group generated a set of questions that invite further investigation. Utilising the first stage of the Question Formulation Technique, a pedagogical approach designed to stimulate student inquiry, a prominent finding from the workshop that questions emerging from focused inquiry provide a useful set of data in their own right. With an explicit workshop focus on learning analytics interoperability, this paper reports on the emergent issues identified in the workshop and the kinds of questions associated with each issue in the context of current research in the field of learning analytics. The study considers the complexity arising from the fact that data associated with learning is itself becoming a digital learning resource while also enabling analysis of learner behaviours and systems usage.

## Introduction

The increasing amount of data generated in digital learning contexts provides opportunities for a range of stakeholders to benefit from learning analytics (LA), including individuals (teachers, students, administrators, educational designers, and parents) as well as organisations. This burgeoning source of data also presents challenges for the design and deployment of learning analytics systems related to interoperability and privacy as well as changing pedagogical and organisational models. As a consequence, new methodologies and technological tools are necessary to analyse and make sense of these data in order to provide intelligent and personalised scaffolding and services to stakeholders including learners (students and trainees), researchers, educators (teachers, professors, and trainers), organisations (schools, universities, professional associations, education and training providers), and administrators, as well as parents and guardians. From an educational perspective, it also seems imperative that appropriate pedagogical and organisational models also inform the design and deployment of learning analytics systems to ensure productive learning and teaching as well as enable quality review. Academic analytics has typically been carried out by the management in universities through processes monitoring registration, retention, graduation, and other parameters that together indicate success or failure. Learning analytics extends this scope with a stronger focus on the actionable results of the analysis and through making use of an increasing variety and volume of data associated with learner activities (Cope and Kalantzis 2014; Cooper 2012). What is new is the extent to which the analysis and its implications could be part of the micro-management of an individual’s learning process. Access to and representation of meaning “– the process of encountering things-to-be-known and representing what one has come-to-know – is increasingly mediated by networked, digital information and communications systems (…) these systems can and often do incidentally record everything that is happening” (Cope & Kalantzis 2014). In this new situation, it is essential to know more about what the basis of any actions might be as a consequence of learning analytics and for what reason the analysis might have been pursued.

Learning analytics starts with data, and access to data from different sources raises a number of concerns related to data sharing and interoperability, protection of privacy for individuals, and protection of business interests for institutions. Thus, the explicit objective of the first workshop on learning analytics held within the annual International Conference on Computers in Education (ICCE) events was to gather researchers as well as stakeholders, including educational technologists and practitioners involved in the analysis and deployment process, and to increase awareness of learning analytics in the Asia-Pacific Society for Computers in Education (APSCE) community^1^. With the strong attendance and active contributions by the participants, the workshop organisers believed this primary objective was met. A second, implicit objective of the workshop was the collection of raw data in the form of open research questions that might inform and help focus ongoing research. These questions provide the data that is the focus of analysis in this paper.

Expressing issues as questions can be a useful way of making some of the complexities of learning analytics more concrete, as Sclater (2014) points out in the literature review on legal and ethical issues of LA (published by UK Jisc at the same time the ICCE workshop was held). Sclater extracted 93 questions from 86 publications, more than a third of them published during 2013–2014. The main areas of concern identified were awareness, consent, ownership, control, the obligations to act, interventions, triage, and the impacts on student behaviour (Sclater 2014). Guided by these questions and the common issues that they share, Sclater carried out an analysis of issues related to codes of practice associated with LA and found that they clustered around 20 issues or topics. This foundational work provides an initial comprehensive thematic grid for discourse around ethical issues related to LA.

While reporting on the outcomes of the first ICCE learning analytics workshop, this paper further brings into focus the emergent issues of concern within an explicit framework of questioning. It has been timely that contemporaneous work has been conducted elsewhere (Cope & Kalantzis 2014; Sclater 2014; Papamitsiou and Economides 2014; Hoel et al. 2016) providing perspective that validates and extends our own findings. However, *findings* can be expressed at various levels of abstraction, and it is our aim that this work informs the development of an ongoing research agenda in an open, investigative manner. At a high level of abstraction, it is reasonable to assert that this emergent field is characterised by a high degree of complexity given that data is itself becoming a digital learning resource as much as the content it may be associated with, while also enabling analysis of learner behaviours and systems usage—in other words, data associated with learner activities represents a new genre of digital learning resource. Complexity also becomes evident when considering the scope of the issues and questions reported on, which is an evolving scope that spans systems design and data governance as well as ethical and privacy concerns.

This paper first provides details of our research methods followed by a report that summarises the content of each paper presented at the workshop, highlighting the key issues and questions. Subsequent sections identify other relevant research, our findings, and analysis. As such, this paper aims to contribute to the discourse on design science research while contributing to the formation of the research agenda, associated with learning analytics concerning the roles of meta-analysis and meta-reflection.

## Context and method

The workshop was designed as a series of paper presentations followed by action research involving audience participation focussing upon generating and collating questions associated with *learning analytics interoperability*. As organisers, we wanted to ensure the workshop achieved a high level of engagement from the participants and provided an opportunity for this beyond listening to a series of paper presentations. This focus upon questions was inspired by the work of Rothstein and Santana (2011) in their exposition of the Question Formulation Technique (QFT) as a process to stimulate student inquiry and questioning skills in a way that is “safe” and non-confrontational. This technique follows a simple sequence of activities that begins with open brainstorming of all possible questions that might be considered relevant to an agreed question focus. A key characteristic of this technique is that a disciplined approach to limiting the activity to question generation is essential—in other words, dialogue concerning plausible answers is not pursued so that an environment of inquiry becomes acute. Apart from developing questioning skills, the QFT has been shown to be an effective approach in workshop settings to engage participants (Rothstein and Santana 2011). While the scope of the workshop was not inclusive of a complete interactive QFT session with participants, it was still used to test the efficacy of the initial stages of the QFT as a means to collect and cluster questions from the participants. During the paper presentations, one of the workshop facilitators also documented questions arising from each paper to further validate the data collection from the focused QFT session that followed all paper presentations. A key motivation for gathering questions at the workshop was to determine, and demonstrate, the viability of questions as a useful data source and specific target for developing a research agenda within the domain of learning analytics.

Closely aligned to the proliferation of data and our research into questions is a growing agenda around twenty-first century skills and competencies focusing on building capabilities in asking the right questions to foster problem solving and critical thinking (Griffin et al. 2012). However, Graesser et al. (2010) have shown that it is more universally the case that “most teachers, tutors, and student peers do not ask a high density of deep questions […] so students have a limited exposure to high-quality inquiry” (p. 125). In the global context where numerous trends are giving shape to a transformation of teaching and learning—trends such as openness, the proliferation of mobile devices, and ubiquitous connectivity—the formulation and articulation of questions therefore represents opportunities and challenges for research that, in turn, may inform innovation and ethical practices relevant in an environment of increasing data ubiquity. Questions, particularly questions exploring rationale and motivation, are what guide educational research and reflective practice. The widespread deployment of new technologies in educational settings raises numerous issues that extend beyond the development of new skills and competencies. It follows that these emerging issues will also propagate many questions—questions that can then be scrutinised, be the subject of detailed analysis, and therefore function as data.

It is important to note here that intrinsic to the QFT is the documentation of questions in the manner that they are first asked. Value judgements or comments as to whether a question is “good” or not are to be avoided because such interventions distract from the inquiry focus and could influence or inhibit the identification or collection of additional questions. Rules such as these also assist in making implementation of the QFT “safe” for students to speak their mind and to pose a question in the terms that they may consider it. Documenting questions as they are asked also provides both authenticity and subsequent opportunity to focus on refinement and/or reformulation (a later stage of activity associated with the QFT). Moreover, in this process further reflection and dialogue help stimulate the inquiry process and refinement of questions (Miller 2014; Rothstein and Santana 2011).

## The papers—key issues and questions arising

Six papers were presented in the workshop. While this is not in any respect enough material for a systematic qualitative review of current research in this area, it does represent a significant collection of perspectives that helped facilitate discussion. Moreover, these papers provided sufficient material for the participants to challenge while also stimulating the participants to explore the questions arising. Contrasted with the results of a recent review by Papamitsiou and Economides (2014) of 40 papers (selected from 208 papers identified by search terms), the ICCE workshop papers identify and raise questions that contribute to the building of an “issues space”, upon which further research can proceed. When classifying research objectives, Papamitsiou and Economides found that the majority of studies in their review investigated issues related to student/student behaviour modelling and prediction of performance, followed by increase in reflection by students and teachers and awareness for the need for improvement following the provision of feedback and assessment services. As the following summary of the workshop papers shows, the initial ICCE workshop issues space appears to be more restricted, although diverse enough to indicate that the ICCE community is in an early phase of making sense of this new field of research. Key issues and questions arising are listed against each paper; however, as the questions were documented during their presentation, questions that had already been documented were typically not repeated. It is important to note that in the context of this investigation, the following summaries are not, however, intended to represent a critique of the papers.

### Preliminary requirements analysis towards an integrated learning analytics system

In the first paper presented, Byung-gi Choi et al. (2014) establish the importance of conducting a requirements analysis in order to construct a united framework for developing an open and extensible learning analytics system. Such an approach is consistent with activity within the domain of international standardisation and in accordance with the theme of the workshop that highlights the role of systems interoperability. The authors report on a reference software architecture they have developed allowing them to identify structure and workflow of learning analytics systems. As participants in a Korean Ministry of Education initiative, the authors make explicit their aim at contributing to the development of international standards supporting worldwide interoperability of learning technology. The paper presents some results of a preliminary requirements analysis towards such an open and interoperable learning analytics system.

Key issues:

The key issues in this paper relate to interoperability and software architectures.

Questions arising:

- What software tools can be used to support learning analytics (LA)?
- What are the dimensions of learning analytics interoperability (LAI)?
- Why is LAI important?
- Who are the stakeholders of LA systems?
- What kinds of issues have been identified as critically important to solve?
- How can requirements of LAI be accurately determined?
- What are the benefits of LA?
- What are the benefits of LAI?
- What needs to be standardised in order to achieve interoperability of LA systems?

### LAI—looking for low-hanging fruits

Hoel and Chen (2014) provided a summary of the current status of LAI and proposed a framework to help structure the interoperability work of requirements analysis and systems scoping. The model is based on a three-dimensional Enterprise Interoperability Framework mapping concerns, interoperability barriers, and potential solutions. The paper also introduces the concept of low-hanging fruits in prioritising analysis and solutions. Data gathered from a small group of Norwegian stakeholders are analysed, and a list of potential interoperability issues is presented.

Key issues:

This paper sets out to find an approach to LA solutions that are complex enough to require that systems interoperate, but simple enough to be implemented without resistance (i.e. being a low-hanging fruit).

Questions arising:

- What are the kinds of systems from which data can be used to support LA?
- Who is doing technical analysis of LA requirements?
- What kinds of commonalities exist in current LA system models?
- What is required in order to create a service from user learning data?
- In what ways do system requirements of LA need to be expressed?
- How can a reference architecture for LAI be expressed?
- In what ways do current frameworks align and differ?

### Making sense of online learning behaviour: a research on learning styles and collaborative learning data

The focus of the study by Sun et al. (2014) is the relationship between learning styles, online behaviours, and group collaborations. Sixty junior students from a university in China using the Sakai course platform were examined to learn about their learning styles. The results revealed a relationship between learning styles and online collaborative behaviour. Nevertheless, the authors conclude that grouping by learning styles might not be the factor affecting group collaborations. Significantly, in framing this paper the authors make explicit four research questions, and these questions served as the ongoing focus of the presentation:

- Which dimensions of learning style have effect on learners’ online behaviours?
- Which kinds of online behaviours could be affected by learning style?
- Is there a significant difference among groups’ online performances?
- Is there a significant relationship between group members’ learning styles and groups’ online collaborative performances?

Key issues:

- “Students with different learning style preferences showed significantly different online behaviors in some patterns.” (Sun et al. 2014, p. 268)
- Correlation of learning style with individual and group performance warrants further research

Questions arising:

- Why might learning styles be significant for LA?x

### How can learning analytics fit into a general evaluation framework?

Stracke (2014) proposes the use of a generic evaluation framework for impact assessment for the purpose of determining how learning analytics can be addressed and embedded in learning design. The Evaluation Framework for Impact Measurement (EFI) combines internal and external impact assessment and provides a generic evaluation framework for learning analytics. Using the international quality standard ISO/IEC 19796-1, the paper discusses which processes and how a learning design specification can be helpful for the introduction and support of learning analytics.

Key issues:

The paper situates LA in the broader context of quality assurance and learning design, exploring whether LA could benefit from the functional principles derived in those fields.

Questions arising:

- How can LA fit into a general evaluation framework as part of learning design?
- Why do we need LA?
- How do changes in the broader context of the evolution of e-learning impact our thinking about the scope of LA?
- In what ways can quality be defined that are useful to LA?
- How can LA fit within the growing forms of open education?
- In what ways can LA be incorporated into learning design?

### Learning analytics data items on digital textbooks

Tamura (2014) introduces historical perspective in introducing the significance of both learning analytics and digital textbooks within the contemporary domain of technology-enhanced learning and standards development. He proposes a set of data items to be collected in digital textbooks. The proposal is based on conventional LMS-based learning activity analytics and modern tablet PC-based learning. The latter has the advantage to collect more detailed data about learners with use of equipped sensors and logging of the manipulation of materials.

Key issues:

This paper is concerned with understanding the variety of data items that enables LA. If the full range of available data is not considered at the design stage of LA systems, then the promise of optimum interoperability will not be fulfilled.

Questions arising:

- What are LA data items?
- What standardisation work related to LA already exists?
- Is the scope of current standardisation work on LA adequate?
- How can metrics for LA be expressed?

### Learning analytics: an enabler for dropout prediction

Tseng et al. (2014) address a key application of learning analytics, prediction of student learning performances, and risks of dropping out. They collected heterogeneous data from a middle school to develop a model for predicting dropout. This exploratory study concluded that dropout prediction using learning analytics may provide more precise information on identifying at-risk students and factors causing them to be at risk.

Key issues:

This paper explores predictive analytics, one of the sub-fields of application for LA, in order to understand the affordances of the use of data supporting this kind of analytics.

Questions arising:

- How can we make sense of online learning behaviour?
- Which dimensions of learning styles affect learners’ online behaviours?
- Which online behaviours are affected by learning style?
- What analytical methods can be utilised with learner data?
- In what ways do group behaviours impact online learning?
- In what ways do teaching styles impact learning behaviours?
- Can LA successfully predict or identify students at risk of dropping out?
- What kinds of data should be included in student records?

## Connecting with current research

Papamitsiou and Economides (2014) found four distinct major axes of current Learning Analytics and Educational Data Mining (LA/EDM) research: *pedagogy-oriented issues* (e.g. student modelling, prediction of performance, assessment and feedback, reflection and awareness); *contextualisation of learning* (e.g. multimodality, mobility—some studies gathered data from the learning context itself); *networked learning* (e.g. MOOCs, social learning platforms); and *educational resource handling* (e.g. getting suggestions for follow-up reading). In Table 1, these axes are contextualised with three dimensions addressing the type of barriers met when pursuing the aims/foci identified in the workshop papers being classified. These dimensions of conceptual, technological, and organisational barriers are used in the paper of Hoel and Chen (2014) and are a refinement of the categories used in enterprise interoperability analysis (Chen and Daclin 2006). Presenting the six papers this way demonstrates clearly that the scope of the workshop papers can be seen as populating approximately 50 % of the range of topics identified by Papamitsiou and Economides (2014).

**Table 1:** Focus of workshop papers—following Papamitsiou & Economides (2014)

Aim/focus of workshop paper	Conceptual	Technological	Organisational
Pedagogy-oriented issues	Stracke (2014)	Sun et al. (2014)	Tseng et al. (2014)
Contextualisation of learning	Hoel & Chen (2014) Tamura (2014)	Byung-gi Choi et al. (2014) Tseng et al. (2014)	
Networked learning			
Educational resource handling		Tamura (2014)	

Furthermore, as Papamitsiou and Economides (2014) explain, “these four axes are not completely autonomous, since significant overlaps may occur”. Nevertheless, when combined with the barrier dimensions, the classification of the six workshop papers—while also noting the call for papers was very open and non-directive—a distinct picture of the research focus of the group attending ICCE 2014 emerges: the focus is split between the question of how learning analytics could impact pedagogy, at different levels of abstraction, on the one side, and, on the other side, how learning is contextualised as a number of different data sources are made available for learning analytics. Patterns within the social aspects of learning is not a theme identified in the workshop papers, and educational resource handling is only present as a distinct focus in one paper.

Table 1 also summarises the foci of the papers in a broad sense. The facilitator questions gathered during presentation and the questions generated from the workshop shown in Table 2 provide a more detailed view of the research interest of this community.

**Table 2:** Questions solicited during presentations and workshop discussions

Facilitator questions gathered during presentations	
#	Question
W1	What are the range of software tools that can be used to support LA?
W2	Why is learning analytics interoperability (LAI) important?
W3	Who are the stakeholders of LA systems?
W4	What kinds of issues have been identified as critical important to be solved?
W5	What are the dimensions of LAI?
W6	How can requirements of LAI be accurately determined?
W7	What are the benefits of LA?
W8	What are the benefits of LAI?
W9	What needs to be standardised in order to achieve interoperability of LA systems?
W10	What are the kinds of systems from which data can be used to support LA?
W11	Who is doing technical analysis of LA requirements?
W12	What kinds of commonalities exist in current LA system models?
W13	What is required in order to create a service from user learning data?
W14	In what ways do system requirements of LA need to be expressed?
W15	How can a reference architecture for LAI be expressed?
W16	In what ways do current LA frameworks align and differ?
W17	How can LA fit into a general evaluation framework as part of learning design?
W18	Why do we need LA?
W19	How do changes in the broader context of the evolution of e-learning impact our thinking about the scope of LA?
W20	In what ways can quality be defined that is useful for LA?
W21	How can LA fit within the growing forms of open education?
W22	In what ways can LA be incorporated into learning design?
W23	What are LA data items?
W24	What standardisation work related to LA already exists?
W25	Is the scope of current standardisation work on LA adequate?
W26	How can metrics for LA be expressed?
W27	How can we make sense of online learning behaviour?
W28	Which dimensions of learning styles affect learners’ online behaviours?
W29	Which online behaviours are affected by learning style?
W30	What analytical methods can be utilised with learner data?
W31	In what ways do group behaviours impact online learning?
W32	In what ways do teaching styles impact learning behaviours?
W33	Can LA successfully predict or identify students at risk of dropping out?
W34	What kinds of data should be included in student records?
Questions generated from workshop	
#	Question
F1	How can we capture learners’ process in ubiquitous learning and doing analytics of these processes?
F2	What is the difference between institutional research and learning analytics?
F3	Can we define and characterise a productive computer-supported learning
F4	How do we capture students’ emotional responses in data?
F5	Who judges the value of the data?
F6	Who owns and controls the data?
F7	Can students and parents control their data?
F8	Is data neutral?
F9	Do we need standards for learning analytics (LA)?
F10	What is the target of learning analysing?
F11	Can an analysis of informal learning process improve learning outcomes?
F12	Is LA an alternative for computer-supported collaborative learning?
F13	How can we combine LA and legacy systems?
F14	What are the ethical issues within LA?
F15	How can we update or keep consistency of learning data?
F16	What do institutions have to do to benefit from LA?
F17	Do we need interoperability or not?
F18	Who should prepare the data: the tool developers or the institution?
F19	How can we identify data clearing house in terms of data auditing?
F20	How can we take care of or support privacy and accessibility?
F21	Is the design of the learning process important?
F22	What is learning?
F23	How can we handle the cultural differences in LET?
F24	How can teachers and parents intervene in learning processes?
F25	How do we sell LA?
F26	Which methods for LA can we trust and why?

In one of the workshop papers, Hoel and Chen (2014) constructed a learning analytics “problem space” based on stakeholder interviews. The same barrier dimensions used in Table 1 were applied, and the following concerns or barriers were extracted:

1. Privacy, trust, and control of data
2. LA affordances and application domains (related to strategies for policy development and implementation for institutions, sectors, and governments)
3. LA context and learning activities (e.g. lack of linkage between learning activity streams and their pedagogical contexts)
4. Legacy system interoperability—information model for LA data exchange
5. LA implementation best practice

Table 3 uses this classification with one additional category for multi-dimensional questions and shows how the audience and facilitator questions fall in these categories.

With 34 questions solicited during presentations and 26 questions from the discussion, there is an about even distribution among the categories with one clear exception, issues related to privacy and ethics were only raised in the workshop discussion session. This is an interesting observation that is discussed in the next section reflecting on the findings of this study.

**Table 3:** Classification of questions by audience and facilitator

Category	Questions by participants	Questions by facilitator
Ethical issues and privacy	6, 7, 14, 20, 26	
LA affordance and application domain	10, 11, 12	7, 18, 22, 27, 28, 29, 31, 32, 33
LA context and activities	1, 3, 22, 23, 24	3, 23, 34
Legacy system and interoperability (data model)	9, 13, 15, 17	2, 8, 9, 10, 15, 16, 17, 24, 25
LA implementation best practice	16, 18, 19, 21, 25	1, 6, 11, 12, 13, 14, 26, 30
Multi-dimensional questions	2, 4, 5, 8	4, 5, 19, 20, 21

## Findings and discussion

The findings of this study relate to both the methodology used and the contributions of the papers and participants to the ICCE 2014 workshop on learning analytics.

### Findings related to the efficacy of the QFT

Given the input, we were able to receive in the short amount of time allocated for one workshop the implementation of the first stage of the QFT as a mechanism for focusing a questioning session while also serving the goal of collecting questions as an output has been demonstrated.

This study shows that the non-biased questioning method easily results in a large number of questions, which in turn leads to new questions—questions begetting questions, rather than questions summoning answers. Moreover, the consequent questions also raise issues of how to sort and evaluate the results and make sense of the collections of the questions as gathered data. The long lists of questions can soon become difficult to handle without a clear strategy on what could be done next or how best to analyse them. Because the QFT is principally used as a means to develop inquiry skills for students, its subsequent stages that follow brainstorming involve classifying the questions as either open and closed. This task is done to help provide insight into which kinds of questions lend themselves to straightforward answers and which promote further inquiry. In subsequent stages, authors of the QFT recommend tasks that change all closed questions into open questions and vice versa followed by group negotiation that might rank the questions for ongoing research or inquiry. Thus, apart from these fairly trivial exercises, the QFT does not provide an explicit strategy for deep analysis as such. This is not a failing of the QFT in our study, however, because we have only utilised it principally as a means to gather the required data. If the method is to be used in further workshop sessions like the one in this study, it may be useful consider ways to augment the process so that deeper analysis can proceed within the group session.

What the collection of questions gathered during the QFT session does demonstrate is validation of the kinds of questions that have been and are being asked by stakeholders and documented elsewhere in the learning analytics literature. The meta approach chosen in this study has been to seek classification schemes in relevant literature, in particular within the papers themselves that were subject to analysis. This might turn out to be an efficient way to bring the questioning to a next level, as papers and presentations that are used to frame discussions often have analytical schemas that only become apparent through meta-analysis.

### Findings following analysis of all workshop papers, presentations, and discussion

Applying the two classifications in Tables 1 and 3 while analysing the questions leads to a hypothesis on where the main research agenda of this community is situated at the end of 2014 and to an interesting discrepancy between questions presented and questions discussed. First, the workshop papers focus on issues related to pedagogical motivation for learning analytics, and to a certain degree, to exploring the space for construction of new LA solutions looking at the contexts of application. It is also clear that the barriers to be overcome at this stage are mainly conceptual and to a certain degree of a technical nature (Table 1). Implementation issues are not yet widely on the research agenda, as it is still early days for learning analytics as a new technology and practice. This finding is in accordance with other recent research. The literature review of Sclater (2014) on legal and ethical issues of learning analytics mentioned in the “Introduction” section provides the rationale for why a code of practice needs to be developed: “Current legal and ethical guidelines have not caught up with innovations in the identification of patterns and new knowledge emerging from the vast datasets being accumulated by institutions” (p. 5).

The “big” questions need to be addressed before solutions can be suggested and tested, as also shown by Dawson et al. (2014). These researchers undertook a citation network analysis of the contribution to the LAK conferences, the large community for LA researchers, and found that “both the conference and the journal special issues are dominated by the lack of conventional research methods and that the authors, regardless of the home discipline, mainly contribute proposal solutions to the conference” (Dawson et al. 2014). Also the European Learning Analytics and Community Exchange (LACE 2015) project^2^ running the Learning Analytics Evidence Hub has found that there are not yet sufficient hard evidence for the efficacy of learning analytics used in schools, universities, and further education (LACE 2015). There is certainly a difference between analytics used for the so-called data-driven classrooms in school education and formative assessment practices in higher education, often deployed under the guise of student retention strategies (Mertier 2014).

Second, the absence of issues related to privacy and ethics in the questions solicited from the workshop presentations and the presence of these issues in the audience’s questions (Table 3) point to a possible gap between design and implementation of learning analytics solutions. There are currently very few large-scale implementations of LA, while the research agenda of the LA research community is focused on what Dawson et al. (2014) have termed “proposal solutions”. From our study, we see that the focus of interest is very much centred around exploring the pedagogic affordances of LA solutions and situating them in a broader educational ecosystem.

## Conclusions and future work

This study has explored how questions can be gathered into collections and then considered as data—or, more precisely, how data about questions, their structure and content, and the context in which they are asked can be collected in order to promote meta-analysis of workshop proceedings and activities within a specific field of research. Within an emergent field such as learning analytics, this seems crucial to spur meta-reflection on the development of the professional discourse. Questioning and using questions as data is an economical way of adding value to the already ongoing activity of further developing a research agenda for the field. On the other hand, it is also the case that a number of questions asked during the workshop are the same kinds of questions asked elsewhere about this field. However, this study has shown that there is a need to bring such an initial exploration to a next level by finding a viable approach to how questions collected as data may then be the subject of detailed scrutiny and analysis. In this paper a couple of approaches have been demonstrated with only preliminary or indicative results being achieved. In order to build on these findings, there is a need to gather more questions as data to identify strategies for deeper investigations such as structural and semantic analysis.

One key feature of questioning in relation to learning analytics that has not been raised in this paper, or in the workshop itself, is an aspect that is so self-evident that it justifies a comment. Questions may also be used as data for learning analytics per se. In simple terms, being able to track the number, form, and quality of questions a student asks may assist in indicating to what depth a topic is being scrutinised.

Arguably, a more challenging frontier associated with learning analytics at this stage of development is that activities and events producing data (about the learner, the learning platform, the learning content, etc.) are also collectively functioning as a new genre of learning resource. Such reflexivity brings a new locus to digital learning that is quite distinct from traditional learning content. Thus, the QFT and other ways of soliciting questions as data could feed into learning analytics cycles that could make learning more reflective and supportive of meta-cognition. This aspect of questioning warrants a research activity of its own. The authors of this paper are involved in work that will lead to more workshops, community exchange, and awareness raising within this particular topic domain of learning analytics. Systematic gathering and analysis of questions resulting from community activity has proven so interesting that the authors plan to build on this work in future ICCE conferences and other settings.

## Abbreviations

APSCE: Asia-Pacific Society for Computers in Education
ICCE: International Conference on Computers in Education
LA: learning analytics
LACE: Learning Analytics and Community Exchange
LAI: learning analytics interoperability
LET: Learning, Education, and Training
QFT: Question Formulation Technique

## Footnotes

1
*More information on the Asia-Pacific Society for Computers in Education can be found at* *www.apsce.net/*

2
*www.laceproject.eu*
